# Using the World Health Organization health system building blocks through survey of healthcare professionals to determine the performance of public healthcare facilities

**DOI:** 10.1186/s13690-017-0221-9

**Published:** 2017-08-31

**Authors:** Tsegahun Manyazewal

**Affiliations:** Department of Health Studies, College of Human Science, University of South Africa, Pretoria, P.O. BOX 392 South Africa

**Keywords:** World Health Organization, Health system, Health system strengthening, Health development goals, Ethiopia

## Abstract

**Background:**

Acknowledging the health system strengthening agenda, the World Health Organization (WHO) has formulated a health systems framework that describes health systems in terms of six building blocks. This study aimed to determine the current status of the six WHO health system building blocks in public healthcare facilities in Ethiopia.

**Methods:**

A quantitative, cross-sectional study was conducted in five public hospitals in central Ethiopia which were in a post-reform period. A self-administered, structured questionnaire which covered the WHO’s six health system building blocks was used to collect data on healthcare professionals who consented. Data was analyzed using IBM SPSS version 20.

**Results:**

The overall performance of the public hospitals was 60% when weighed against the WHO building blocks which, in this procedure, needed a minimum of 80% score. For each building block, performance scores were: information 53%, health workforce 55%, medical products and technologies 58%, leadership and governance 61%, healthcare financing 62%, and service delivery 69%. There existed a significant difference in performance among the hospitals (*p* < .001).

**Conclusion:**

The study proved that the WHO’s health system building blocks are useful for assessing the process of strengthening health systems in Ethiopia. The six blocks allow identifying different improvement opportunities in each one of the hospitals. There was no contradiction between the indicators of the WHO building blocks and the health sustainable development goal (SDG) objectives. However, such SDG objectives should not be a substitute for strategies to strengthen health systems.

## Background

The 2016 transition in global health from Millennium Development Goals (MDGs) to Sustainable Development Goals (SDGs) is a remarkable move for resource-limited countries that have been struggling to improve the quality of healthcare at the ground [1–4]. The various MDG targets for health were instrumental in shaping healthcare outcomes; with a significant number of resource-limited countries able to meet the targets [5, 6]. Similarly, it is worthy that these countries have given increased attention to the current SDGs for health. However, fulfillment of global health targets, unaided by the overall health system strengthening efforts which is mainly a national issue [7], does not guarantee improvement of the overall health system [8–10].

**Fig. 1:**
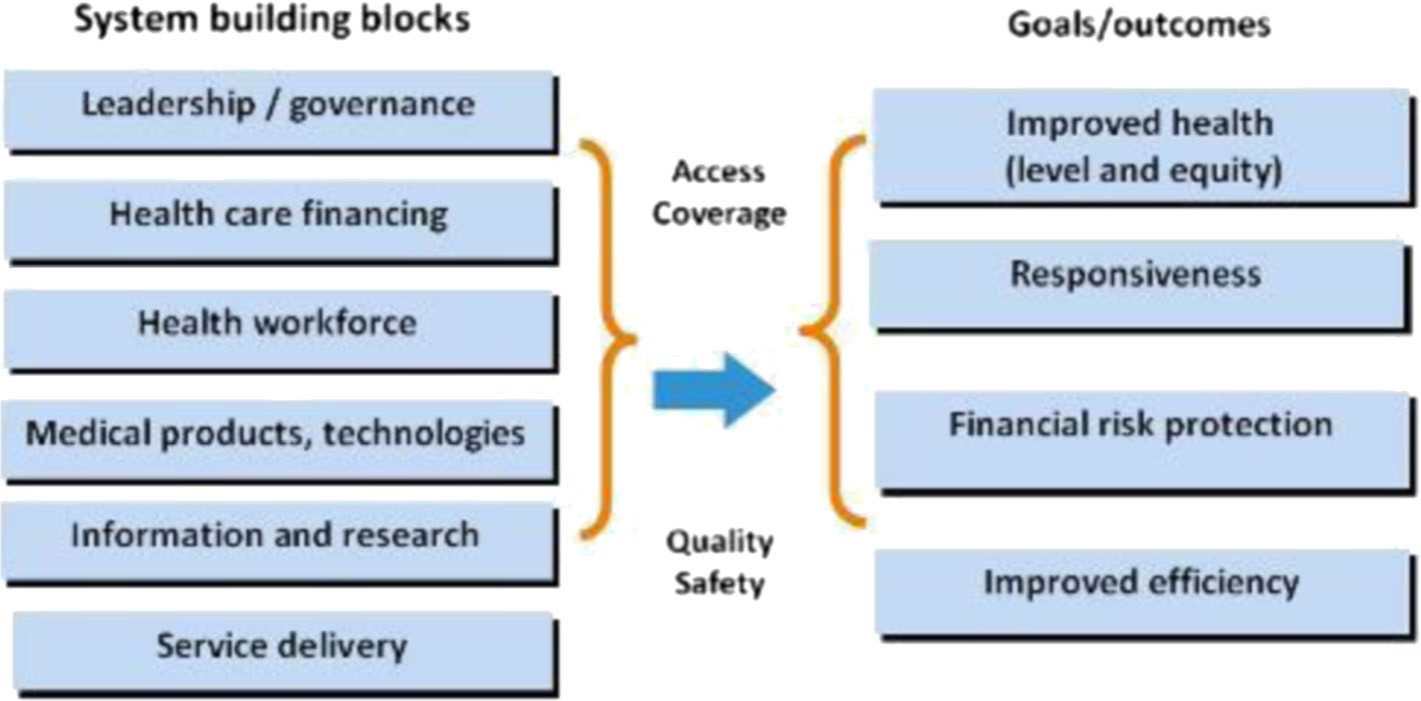
The WHO Health Systems Framework [11]

Acknowledging the health system strengthening agenda, the World Health Organization (WHO) has formulated a health systems framework that describes health systems in terms of six building blocks which include service delivery, health workforce, information, medical products, vaccines and technologies, financing, and leadership/governance (Fig. 1) [11]. Good *service deliveries* are those which deliver effective, safe, quality personal and non-personal health interventions to those that need them, when and where needed, with minimum waste of resources. A well-performing *health workforce* is one that works in responsive ways, fair and efficient to achieve the best health outcomes possible, given available resources and circumstances. A well-functioning *health information* system is one that ensures the production, analysis, dissemination and use of reliable and timely information on health determinants, health system performance and health status. A well-functioning health system ensures equitable access to essential *medical products, vaccines and technologies* of assured quality, safety, efficacy and cost-effectiveness, with scientifically sound and cost-effective use. A good *health financing* system raises adequate funds for health, in ways that ensure people can use needed services and are protected from financial catastrophe or impoverishment associated with having to pay for them. *Leadership and governance* involve ensuring the existence of policy frameworks combined with effective oversight, coalition building, regulation, attention to system design and accountability. Strengthening health system means improving these six health system building blocks and managing their interactions in ways that achieve more equitable and sustained improvements across health services and health outcomes which require technical and political knowledge and action [11]. The WHO has supported its health system framework with a monitoring and evaluation framework to monitor program management of health system investments, assess health system performance and evaluate the results of health reform investments [12].

Studies indicate the WHO health system framework is instrumental in strengthening the overall health system and uses as catalyst for achieving global health targets such as the SDGs. Unlike other health system strengthening strategies which are disease-specific [13] or narrow [14–16], the WHO’s health system framework intends to improve the overall health in a responsive, financially fair and most efficient way [11]. Evidences revealed that the framework helps to assess in-country healthcare performances [17], interactions between health reforms and country health systems [18], implications of health sector reforms [19], and the status of health facilities [20] and specific health problems [21].

The various global health development goals and commitments have been the alarm bells to the government of Ethiopia. With significant contributions from global partners, the government of Ethiopia tailored to meet many of the global health indicators. For instance, the country has successfully achieved six of the eight MDGs, including MDG Goal 4 and other targets for HIV/AIDs, malaria, tuberculosis and other diseases, while MDG 5 (improve maternal health) is an area the country was off-track [22]. However, meeting such global targets alone could not justify improved and sustainable health system and the readiness responding effectively to unanticipated health threats.

Since 2008, the Ethiopian government is implementing healthcare reform aimed at strengthening the overall health system [23]. A system of tracking clients’ opinions and complaints about services has been put in place to enable the Ethiopian government to take appropriate and immediate actions. Based on the principle of BPR [24], the FMoH has conducted “as is” analysis to document pertinent issues, understand the pros and cons of the existing healthcare delivery system in the country and figure out the different dynamics that should be considered in the redesign of the new reform. With these, “to be” business processes were designed, public health sector standards formulated, and standard operating procedures and implementation tools developed [25]. The reform has been progressively implemented through a series of training sessions for managers and technicians at all levels followed by changes in staff deployment, specific job assignments and the recruitment of new staff. Stretched objectives were synthesized and sub processes that form the core process identified. Public hospital services were structured into three major case teams namely; Emergency, Outpatient, and Inpatient, where Outpatient and Inpatient case teams were further classified into eight and nine case teams, respectively [26]. However, despite implementing the healthcare in public healthcare facilities, little is known about the current status of the public hospitals in terms of the WHO’s health system building blocks.

Building on the lessons learned in the MDGs and considering the current socioeconomic landscape, the Government of Ethiopia is developing and implementing its Health Sector Transformation Plan (HSTP) – a five-year (2015–2020) national health sector strategic plan and the first phase in the ‘Envisioning Ethiopia’s Path towards Universal Health Coverage through Strengthening Primary Healthcare’ [22]. In the HSTP, four pillars of excellence are believed to help the sector attain its mission and vision: health service delivery, quality improvement and assurance, leadership and governance, and health system capacity. The four pillars are linked with the WHO’s six health system building blocks.

The status of the WHO’s six building blocks at public health facilities would configure the health system and its outcomes in national as well as global needs. Public hospitals, with their possibilities for improved healthcare services, training, research and innovations, are potential to favorably influence the broader array of the healthcare system. Thus, periodic evaluation of the overall healthcare system is required to identify gaps and provide appropriate interventions. Such constructive insights would consolidate global health development goals and routine healthcare needs for possible improvements of the overall health system. Keeping this up-front, this study intends to determine the current status of the WHO’s six health system building blocks in public hospitals in central Ethiopia.

## Methods

A quantitative, cross-sectional study was carried out to analyze the status of the Six WHO health system building blocks in public hospitals in central Ethiopia, thus Addis Ababa. Addis Ababa was selected among the 11 administrative divisions of Ethiopia considering its presence as the largest and city capital of Ethiopia. The Addis Ababa Health Bureau has been implementing the healthcare reform in public healthcare sector that it owned. The bureau administers six public hospitals which deliver advanced preventive and curative health services, from which all that have been implementing the BPR healthcare reform since its inception in 2009 (*n* = 5) were purposively selected to maximize the scope of the study thereby ensure external validity. Hence, the five public hospitals were purposively sourced as they were in a post-reform phase.

The study was conducted between January and June 2015. The target group was all healthcare professionals in the public hospitals (*n* = 1681) which included medical doctors, nurses, laboratory professionals, pharmacists, dentists, health officers, and sanitarians. Of these, all who started working in the hospitals at least a year ahead of initiation of the reform (*n* = 476, 28%) were purposively drawn to select respondents who knew the performance of the hospitals before implementation of the reform and better analyze the changes that occurred because of the reform.

A self-administered, structured questionnaire targeting healthcare professionals was developed in a 5-level scale and used for data collection. To elicit a quality questionnaire, secondary data and related studies conduct elsewhere were reviewed and the questionnaire pretested. The questionnaire was structured to cover the six WHO health system building blocks and specific indicators were adapted from the target in the BPR healthcare reform document for improvements in healthcare facilities:

- *Leadership/governance*: new organizational practices and policies, the best use of resources, appropriate use of staff working hour, satisfaction of patients and providers, capacity to assemble and manage resources;
- *Healthcare financing*: efficient and effective healthcare financing system, linkage of financial mobilization with evidence-based plan, effective budget consumption, the required financial resources to ensure sustainability, and reduced wastage and enhanced cost-effective interventions;
- *Health workforce*: qualified staff, job satisfaction, motivation, conducive structure, appropriate and timely feedback;
- *Medical products/technologies*: adequate drugs, medical supplies, medical apparatuses and equipment, up-to-date technologies for patient diagnosis, new organizational practices and policies, networking with the external environment;
- *Information*: monitoring and evaluation, up-to-date and appropriate guidelines and protocols, appropriate internet access, easy and time-efficient reporting system;
- *Service delivery*: patient satisfaction, on-time services, improved treatment and respect to patients, patient indiscrimination, mode of communications suitable to patients.

Data analysis was performed through calculation of descriptive statistical procedures on IBM SPSS version 20. Each of the five responses had a numerical value (1–5), in which the highest two scoring answers (4 and 5) were taken as positive outcomes while the rest three responses were considered as negative outcomes. With this, a positive outcome for each of the WHO six building blocks had a value of 4, which is equivalent to a mean percentage score [27] of 80% or above. As the questions were grouped under the six building blocks, a scale score was computed as the mean of the scale item scores, while the median score was employed to measure central tendency among individual questions.

The study was granted ethical clearance certificate from the Higher Degrees Committee of the Department of Health Studies, University of South Africa and the Research and Technology Transfer Core-process of the Addis Ababa City Administration Health Bureau. Informed consent form was developed for each respondent to read and sign before moving on to fill-in the questionnaires.

## Results

### Socio-demographic profile

A total of 406 healthcare professionals participated in the study, among which 282 (69.5%) were women. The majority of participants (195, 48%) were in the age ranging from 30 to 39, while very few (26, 6.4%) were in the age ranging from 50 to 59. The largest proportion of participants (304, 74.9%) was nurses, followed by medical doctors (35, 8.6%), medical laboratory professionals (24, 5.9%), pharmacist (16, 3.9%), X-ray technicians (11, 2.7%) and sanitarians (2, 0.5%). Academically, the largest proportion of participants (342, 84.2%) had bachelor’s degree, followed by Diploma (37, 9.1%), medical doctorate with specialization (18, 4.4%), MSc/MA or MPH (7, 1.7%), and certificate 2 (0.5%). A large number of participants (202, 49.8%) worked as a healthcare professional for 10 to 19 years.

Table 1 summarizes the status of the WHO six building blocks in the study hospitals.

**Table 1:** The status of the WHO health system building blocks in public hospitals

WHO health system building blocks	Score (%)
Leadership/Governance	
New organizational practices and policies	60
The best use of resources	65
Appropriate use of staff working hour	58
Satisfaction of patients and providers	61
Capacity to assemble and manage resources	61
Average	**61**
Healthcare financing	
Efficient and effective health care financing system	63
Linkage of financial mobilization with evidence-based plan	58
Effective budget consumption	62
The required financial resources to insure sustainability	65
Reduced wastage and enhanced cost-effective interventions	61
Average	**62**
Health workforce	
The required qualified staff	63
Job satisfied staff	51
Motivated staff	49
Conducive staff rest room	47
Conducive structure	62
Appropriate and timey feedback	56
Average	**55**
Medical technology	
Enough drugs, medical supplies, medical apparatuses and equipment	56
Up-to-date technologies for patient diagnosis	58
New organizational practices and policies	56
Networking with external environment	60
Average	**58**
Information	
Monitoring and evaluation system	64
Up-to-date and appropriate guidelines and protocols	57
Internet access	38
Easy and time-efficient reporting system	53
Average	**53**
Service delivery	
Satisfied patients	63
On-time services	69
Improved treatment and respect to patients	66
No patient discrimination	80
Services to patients using mode of communication suitable to patients	69
Average	**69**

### Leadership/governance building block

Data was collected about elements of governance/leadership from public healthcare perspectives. Given the increasing diffusion of new organizational practices and policies across the study hospitals, the impact of this practice had little benefit (60%) when weighed against the perceived value (≥ 80%). Resource management had challenges in that the best use of resource (65%) and the capacity to assemble and manage resources (61%) in the hospitals were not adequate, signifying that leadership skills were loosen on this matter. Regarding staff working hour, the result was smaller (58%) and even lesser than all the rest scores in leadership/governance section, implying a higher misuse of working hours in the hospitals. Healthcare professionals in the hospitals perceive an overall 61% satisfaction of employees and patients regarding how the hospitals are currently operating. In this leadership/governance category, a relatively highest score (65%) was in the use of resources, while the least score (58%) was in the use of staff working hours. In general, the average score of leadership/governance in this study was 61%, which was lower than the 80% score perceived in the WHO’s health system framework.

### Healthcare financing

For this building block, five questions were posed to realize the healthcare financing capacity of the hospitals. The author learned from the healthcare professionals that there is a loosen linkage between financial mobilization and evidence-based planning. The performance of the hospitals toward reducing wastage of resources while enhancing cost-effective interventions was deprived and the overall budget consumption was ineffective, which were potential for financial recessions. There had been a relatively better achievement (65%) towards gaining financial resources essential for sustainability of healthcare services. The average score of leadership/governance was 62%, which was yet lower than the perceived 80% score.

### Health workforce

This section witnessed multifactorial public health workforce challenges which need contextual changes in line with the WHO health workforce needs. The study captures major claims on availability of staff restrooms (47%). There exist job dissatisfaction and demotivation of the public health workforce, with potential impacts on the overall health system. The average score for health workforce was 55%, which was much lower than the 80% score perceived for the WHO’s health system framework for health workforce.

### Medical products/technologies

The impetus for medical technologies and practices had gaps when scaled against the intended WHO requirements. The hospitals are not well-resourced with enough drugs, medical supplies, medical apparatus and equipment. As well, there was a need for networking the hospitals with the external environment for a possible exchange of medical technologies. The average score of medical technologies was 58%, which was much lower than the 80% score intended in the WHO’s health system framework for medical products/technologies.

### Information

The study set out to the existing information system in terms of the WHO building block and found that information is limited in opportunity and scope. Healthcare professionals in the hospitals recognized that access to internet is limited (38%) to hospital staff. The hospitals’ duties were poorly supported by up-to-date and appropriate guidelines and protocols. Monitoring and evaluation system of the hospitals was viewed as a fairly practice when compared with other responses in this section. The average score for information was 53%, which was much lower than the intended 80% score.

### Service delivery

Service delivery had some fairly gains. The healthcare professionals (80%) highlighted that there are no patient discriminations, though this finding needs to be confirmed with patients. On-time access to services and availability of services in a mode of communication suitable to patients both scored 69%. The average score for service delivery was 69%, which was yet lower than the perceived 80% and above score. While this 69% score for service delivery was the highest among the six WHO health system building blocks.

### Overall performance

In line with the WHO health system building blocks, the overall health system performance of public hospitals in central Ethiopia was 60%. Looking at each building block, results were lesser for information (53%), health workforce (55%), medical products/technologies (58%), leadership/governance (61%), healthcare financing (62%) and service delivery (69%). There was a significant difference in healthcare performance between at least two hospitals (χ^2^ = 571.902, *p* < .001).

## Discussion

This study aimed to find out the current status of the six WHO health system building blocks in public healthcare facilities in Ethiopia. The findings revealed that the overall performance of public hospitals which were in a post-reform phase was lesser when weighed against the WHO’s six health system building blocks. The public hospitals scored less for each WHO building block, which includes information, health workforce, medical products/technologies, leadership/governance, healthcare financing, and service delivery.

Over the past decade, the WHO and other organizations in the field have given much attention to the issue of health systems strengthening [7, 14, 28–30] and the government of Ethiopia has been in the loop reaffirming its commitments and consolidating the gains [10, 13, 31–33]. This study deepens understanding of how healthcare facilities can develop a platform to assess and monitor their performances for on-going improvements in the context of the WHO’s six health system building blocks. The approach facilitates results measurement through generating data for measuring outputs for a better and sustained improvement. The main data source of the study was from healthcare professionals which are the ultimate resources of health systems and markedly responsible for monitoring the healthcare climate [34]. Studies exhibited that healthcare professionals are key sources of information to track and monitor health system progresses [35, 36], and there have been unwanted variations in healthcare practices that cannot be explained by patients [37].

The study witnessed that there were major issues which may affect the status of the six WHO building blocks in public hospitals. The hospitals’ governances did not pursue staff loyalty to effectively use their time to maximize the hospitals’ capacity and sustainability. This needs keener interest of the hospitals’ administrations to probe on the governance constraints and take possible interventions to attain the intended WHO governance/leadership needs among others.

Healthcare financing was another concern in the current status of the WHO building blocks. The healthcare financing capacity, in relation to healthcare goods and services, indicates that mandatory steps still remain. The hospitals were not effective enough in linking financial mobilization with evidence-based plans. Besides, the hospitals were unable to reduce wastages and enhance cost-effective interventions. These indicate that the healthcare financing gaps identified in this study need to restructure. The policy options that the FMoH proposed to address challenges in healthcare financing are health insurance and social health insurance [38]. These two options could be considered as possible options for improvement in healthcare financing. However, additional specific strategies which target public hospitals need to be formulated to address finance related gaps identified in the study. As households in rural part of Ethiopia are included in public health insurance schemes [39], the Ethiopian government could shift existing budgets for strengthening health system at public hospitals. The cost which was estimated for implementing the HSTP in the year 2015/16–2019/20 is $177,723,169, where the highest share goes to human resource and infrastructure [22]. From this budget, public hospitals need to secure a significant share to enhance their capacity and be equipped with innovative technologies.

Regarding health workforce, only a fraction of the WHO building block for workforce was achieved. Lack of job satisfaction, motivation, convenient infrastructure, timely performance feedback, and qualified staff were major gaps identified in the public hospitals. This finding is in support of previous studies conducted in Ethiopia at different times and with different target populations [40–43]. A previous report of the FMoH also witnessed scarcity and inappropriate usage of medical products and technologies in public hospitals [22]. There were scarcities of drugs, medical supplies, medical apparatus, and equipment in the hospitals. There was less effort taken towards initiating new practices for effective and advanced usage of technologies. Hence, evocative strategies with huge investments in health workforces’ education and training, management, retention, incentives, motivation, and job satisfaction are required to meet the intended human resource need.

Similarly, information communication and exchange methods, which are active catalysts in the broad-based health development, were stagnated, thus need improvements. The hospitals have been highly affected by lack of sufficient internet access in the hospitals, which could hinder staff from updating their knowledge and translating to their patients. In the same sense, there have been many guidelines that the FMoH and its partner organizations developed and shared for bench-level use. However, the guidelines and other standard operating procedures were not fairly in place as quick references. Such constraints need practicable health information strategies coupled with monitoring system. Overall, there is a need for further progress in all the six WHO health system building blocks. The use of the six WHO health system building blocks was proved to be instrumental for assessing and following-up of the overall healthcare system.

This study is with some limitations. Evidences only from healthcare professionals might be subjected to their experiences and expectations. Hence, expanding the sources of the study data set to capture more inclusive information that could be obtained from patients or the existing hospital data was necessary. On the other side, the relatively low proportion of physicians among those who answered the interview may hide the actual view of clinicians in the analysis. Or else, the study’s approach proved that the use of the six WHO building blocks to numerically assess and monitor public healthcare sectors is possible. Healthcare facilities can employ this approach to assess and follow-up their own strengths and weaknesses with minimum costs. With this, it is possible to strengthen health system at various levels, and simultaneously integrate global health commitments for sustainability and ownership.

## Conclusion

The study proved that the WHO’s six health system building blocks are useful for assessing the process of strengthening health systems in Ethiopia. The six blocks allow identifying different improvement opportunities in each one of the hospitals. There is no contradiction between the indicators of the WHO health systems building blocks and the health sustainable development goal (SDG) objectives. However, such SDG objectives should not be a substitute for strategies to strengthen health systems.

## Abbreviations

FMoH: Federal Democratic Republic of Ethiopia Ministry of Health
HSTP: Health Sector Transformation Plan
MDG: Millennium Development Goal
SDG: Sustainable Development Goal
WHO: World Health Organization
